# A Case of Pericarditis With Pericardial Effusion Due to Lyme Disease

**DOI:** 10.7759/cureus.87476

**Published:** 2025-07-07

**Authors:** Osamede Agho, Sara Tariq, Arshia Ahmed, Salman J Khan, Lela Adeoshun

**Affiliations:** 1 Internal Medicine, Guthrie Lourdes Hospital, Binghamton, USA; 2 Internal Medicine, Igbinedion University Medical School, Benin, NGA; 3 Public Health, University of Massachusetts Amherst, Amherst, USA

**Keywords:** bacterial pericarditis, doxycycline, lyme disease, oral doxycycline, pericardial effusion, pericardiocentesis

## Abstract

Acute pericarditis with pericardial effusion caused by Lyme disease is very rare and requires excluding other more common possible causes of acute pericarditis. Common causes of acute pericarditis include viral and bacterial infections, connective tissue disorders such as rheumatoid arthritis, systemic lupus erythematosus, or scleroderma, or trauma to the heart, such as from a heart attack or heart surgery. While Lyme disease is mainly known for cardiac effects such as heart blocks and arrhythmias, it can also rarely cause pericarditis with pericardial effusion. In this case report, we present a patient with typical manifestations of acute pericarditis with pericardial effusion, and the patient was found to be positive for Lyme disease. Following pericardiocentesis and a three-week course of doxycycline, her symptoms resolved.

## Introduction

Lyme disease, caused by *Borrelia burgdorferi* (*B. burgdorferi*), is the most commonly reported tick-borne illness in the United States. *B. burgdorferi* is commonly transmitted by four species of the Ixodes tick, and transmission occurs when infected ticks attach to humans to take a blood meal. Over 90% of infections in the United States occur in the Northeastern region. Infections in the United States also occur in areas outside the Northeast, for example, northern California. While deer are hosts for the adult ticks, they do not carry the spirochete, *B. burgdorferi*. The primary animal reservoir for *B. burgdorferi* in the United States is the white-footed mouse, which serves as the preferred host for the nymphal and larval forms of the deer tick.

Lyme disease is a multisystem infectious disease with a wide range of manifestations [1]. While its clinical spectrum typically involves dermatological, neurological, and musculoskeletal symptoms, cardiovascular involvement is less common but can be severe. Lyme carditis, for example, is a rare manifestation of the disease, reported in 1.5-10% of cases in North America and 0.5-4% in Europe. Another cardiac manifestation of Lyme disease is pericardial effusion, an accumulation of fluid within the pericardial sac, which may result in pericarditis or even life-threatening cardiac tamponade. Pericardial effusion in Lyme disease is a rare yet significant complication that underscores the importance of considering Lyme disease in the differential diagnosis of unexplained pericardial effusion, especially in endemic regions or in patients with a history of tick exposure [2]. This case report presents a patient with Lyme disease who developed pericarditis with pericardial effusion, highlighting the diagnostic challenges, clinical features, and management strategies associated with this rare manifestation.

## Case presentation

A 69-year-old female patient with a medical history of asthma, factor V Leiden deficiency on Xarelto 20 mg daily, and osteoarthritis presenting with complaints of chest pain, chest tightness, and shortness of breath of four days duration. She described the pain as a dull ache associated with chest tightness as she made a fist, holding it to the center of her chest. She also reported intermittent low-grade fever and chills. She denied cough, sore throat, sinus congestion, or rhinorrhea. She denied any recent upper respiratory infection and stated that there was no improvement in her breathing with the use of her asthma medications. She also noted that the shortness of breath was worse when she lay flat on the bed and improved when she sat up and leaned forward. She denied any history of trauma or exposure to chemotherapy or immunotherapy.

Upon examination, her vital signs revealed a blood pressure of 112/75 mmHg, a heart rate of 93 beats per minute, an oxygen saturation of 95% on room air, a temperature of 37.6°C (97.8°F), and a respiratory rate of 18 breaths per minute. She had reproducible chest pain and mild dyspnea, and the respiratory exam showed decreased breath sounds bilaterally as the patient was unable to take deep breaths. The cardiovascular examination revealed distant heart sounds on auscultation. The rest of the systemic examination was unremarkable. Labs and imaging were ordered the same day she presented to the hospital. Her initial laboratory reports showed a WBC count of 10.29, a CRP of 22.10, and an ESR of 81. Troponin was <0.01.

**Figure 1:**
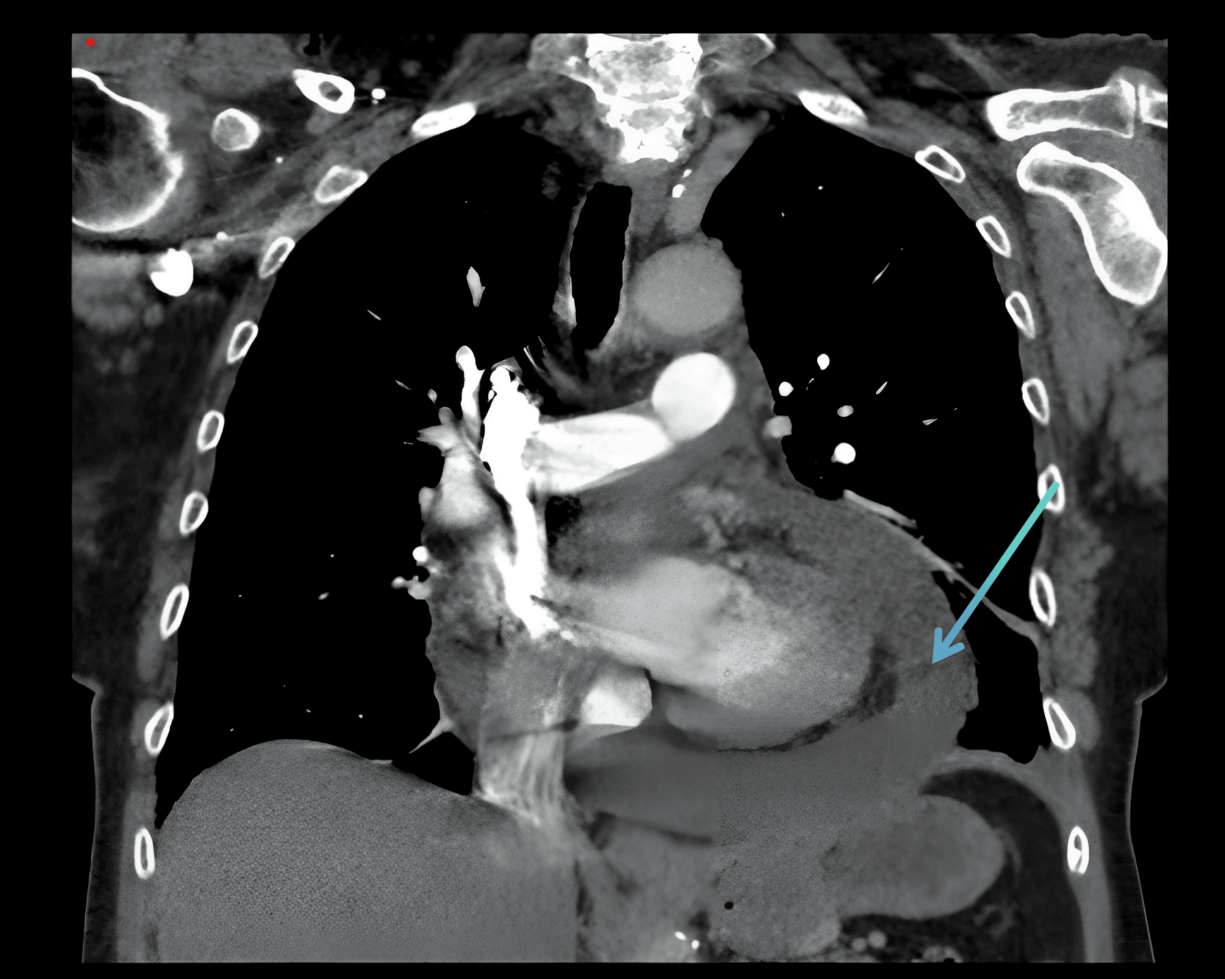
CT angiography chest (coronal view) showing a large circumferential pericardial effusion (blue arrow) CT: computed tomography

**Figure 2:**
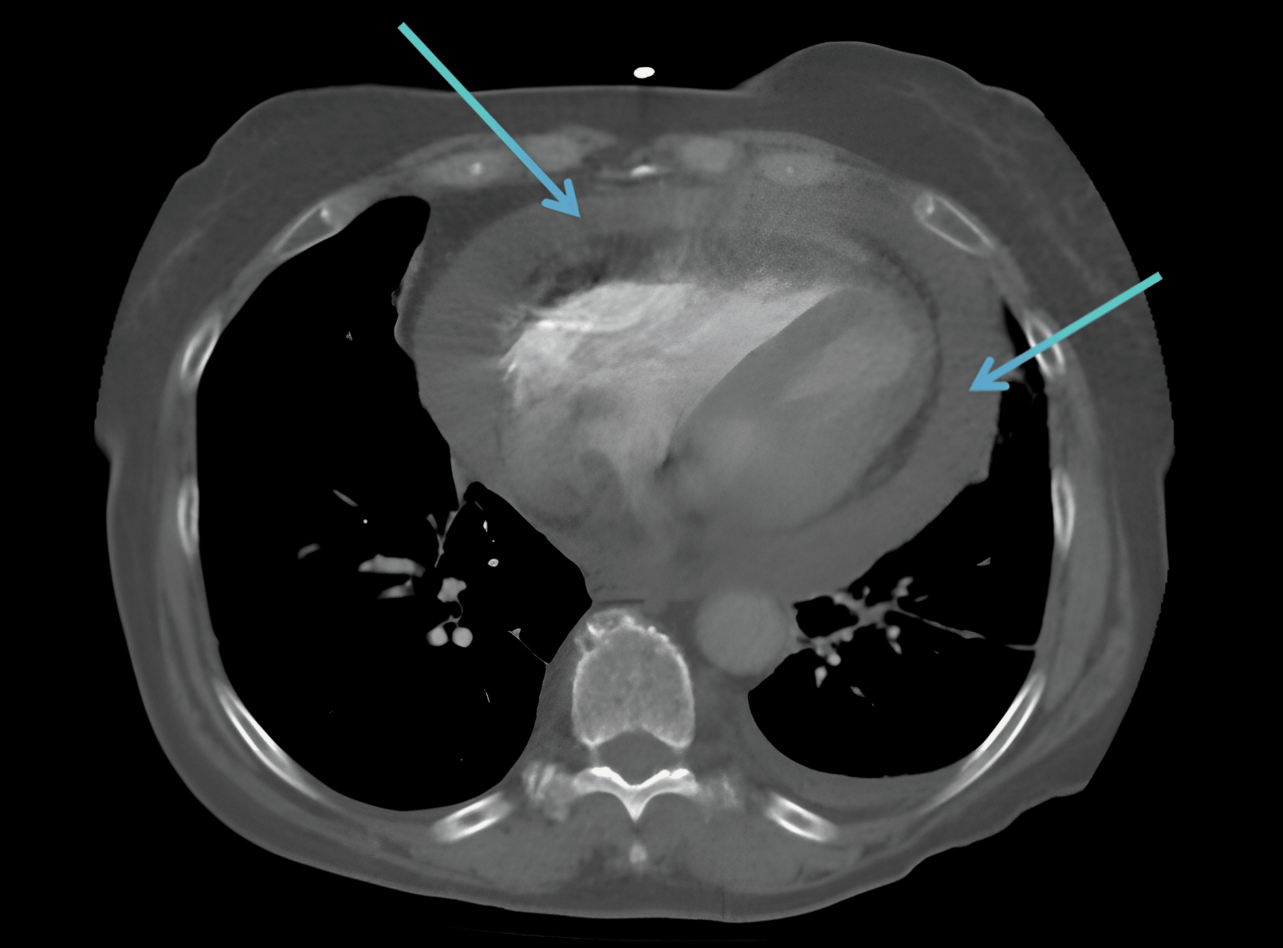
CT angiography chest (axial view) showing a large circumferential pericardial effusion (blue arrows) CT: computed tomography

**Figure 3:**
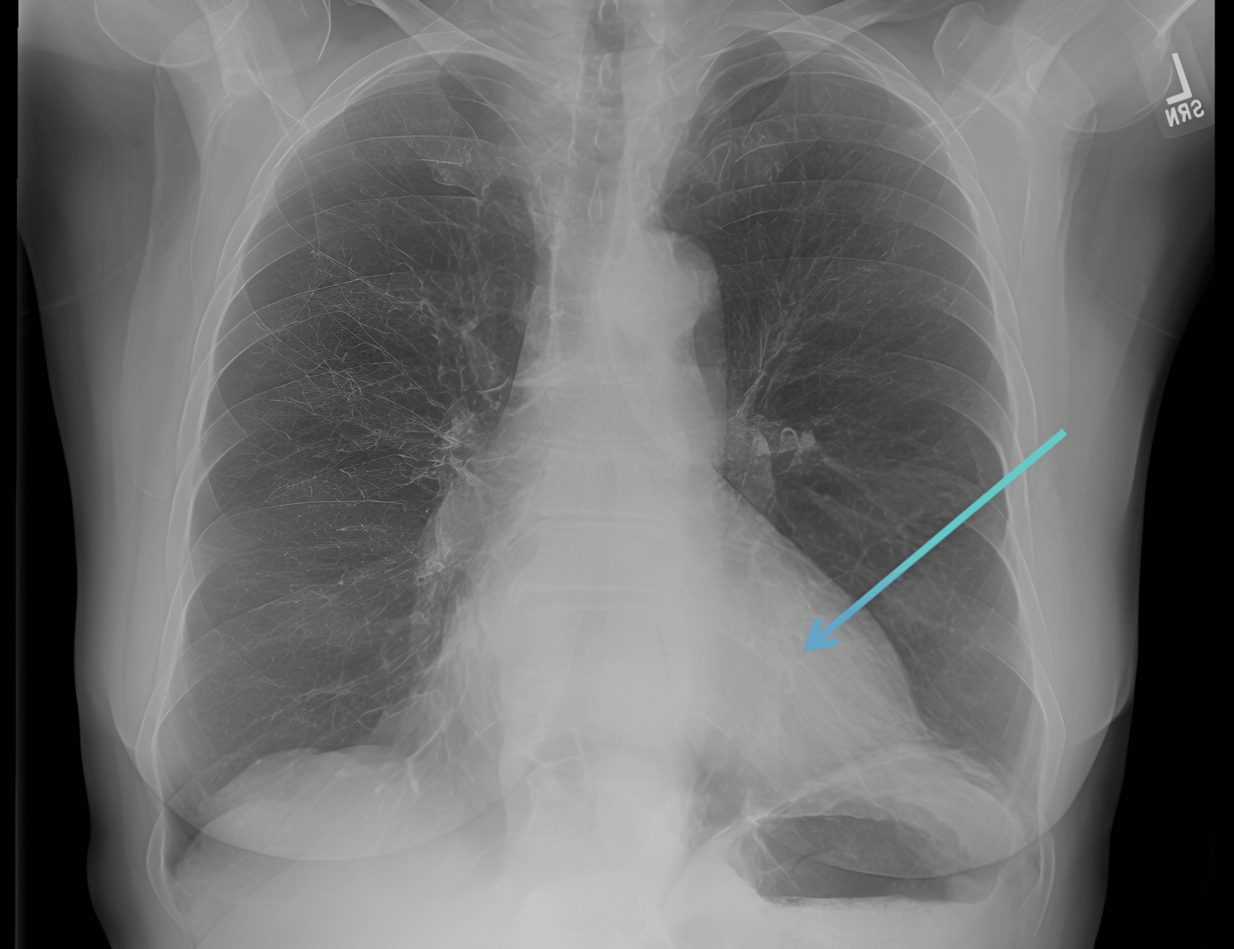
Chest X-ray showing a globular heart (blue arrow)

CT chest coronal and axial views showed a large pericardial effusion (Figures 1-2). Chest X-ray showed cardiomegaly with a globular heart (Figure 3). Due to the rarity of this disease condition, other common causes of Lyme carditis with pericardial effusion were excluded. These include bacterial, viral, autoimmune causes, trauma, uremia, medications, chemotherapy, and immunotherapy. Laboratory investigations, including blood culture, autoimmune screen, viral panel, TSH, and Lyme disease screen, were requested. Blood cultures taken five days after admission showed no growth; pericardial fluid cultures were negative. The TSH level was 1.80, and a comprehensive viral respiratory panel was also negative. ANA testing showed a low titer of 1:80, and EBV testing was suggestive of a past infection. EBV capsid ab IgM was <36, and EBV capsid ab IgG was 144. The Lyme disease antibody screen report was available approximately five days post-admission and was positive. A confirmatory test using Western blot was performed, confirming the presence of *B. burgdorferi*. Lyme disease IgM immunoblot showed reactivity to 2 of 3 IgM-specific *Borrelia* proteins.

Autoimmune and Lyme disease screening results are shown in Table 1. Putting together her clinical presentation, serological testing, and imaging findings, a diagnosis of acute pericarditis with moderate to severe pericardial effusion caused by Lyme infection was made after other possible causes of acute pericarditis were ruled out. Although a concurrent autoimmune process cannot be completely ruled out in this case, the patient responded to treatment with oral doxycycline with resolution of her symptoms and inflammatory markers.

**Table 1:** Quantitative serology and Lyme disease testing ANA: anti-nuclear antibody, Ab: antibody, IgM: immunoglobulin M, *B. burgdorferi*: *Borrelia burgdorferi*

Quantitative serology and Lyme disease testing	
Tests	Results
ANA	Positive
ANA pattern	Cytoplasmic fluorescence pattern
Anti-nuclear Ab titer	1:80
Lyme Ab screen	>1.23
*B. burgdorferi* IgM Ab	Reactive to 2 of 3 IgM proteins

**Figure 4:**
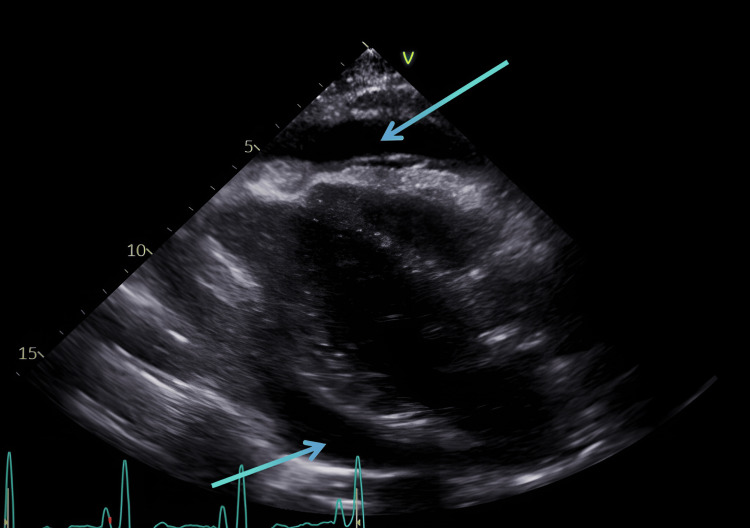
Transthoracic echocardiogram (parasternal long axis view) showing a large circumferential pericardial effusion (blue arrows)

The patient also underwent a transthoracic echocardiogram, and the report revealed a moderate to large free-flowing pericardial effusion without internal echoes (Figure 4). It also showed chamber collapse with right atrium involvement for less than 50% of the cardiac cycle, suggesting tamponade physiology. The patient had pericardiocentesis done and had 450 ml of fluid drained. A transthoracic echocardiogram was done post pericardiocentesis, and the result showed small pericardial effusion. She received a three-week course of oral doxycycline, ibuprofen, and colchicine, which resolved her symptoms. A repeat echocardiogram was performed one month after her post pericardiocentesis echocardiogram, which showed findings unchanged from the previous one. Symptom resolution was reported during her cardiology follow-up visit, two months post pericardiocentesis.

## Discussion

Lyme disease, caused by the bacterium *B. burgdorferi*, is the most common vector-borne illness in North America and Europe, primarily transmitted by the tick Ixodes [1]. It typically presents with a characteristic erythema migrans rash and, if untreated, can progress to affect multiple organ systems, including the skin, joints, nervous system, and, rarely, the heart [1]. Cardiac involvement in Lyme disease, though uncommon, can manifest in a variety of ways, such as Lyme carditis, which typically presents as atrioventricular block and more rarely as pericardial effusion [2,3]. How do I correctly mention figures, tables, videos, and interactive models in the text?

Pericardial effusion in Lyme disease is a rare manifestation, but it is important to consider, as it can lead to severe outcomes, including pericarditis or even life-threatening cardiac tamponade [4,5]. The pathogenesis of pericardial effusion in Lyme disease is thought to result from direct infection of the pericardium by *B. burgdorferi* or, more likely, from an autoimmune or inflammatory response to the organism. Inflammation of the pericardial tissue can lead to fluid accumulation in the pericardial sac. In severe cases, the effusion may cause compression of the heart, resulting in tamponade, a medical emergency [5].

Diagnosing Lyme disease in the setting of pericardial effusion presents several challenges. While Lyme carditis is more frequently associated with conduction disturbances, pericardial effusion may not always be clinically apparent or easily identified. Diagnosis is confirmed through serologic testing for *B. burgdorferi* antibodies, including enzyme-linked immunosorbent assay (ELISA), Western blot, and polymerase chain reaction (PCR) testing. However, PCR for *Borrelia* in pericardial fluid is not universally available [6]. Additionally, imaging modalities such as echocardiography or CT play a pivotal role in detecting the presence and severity of pericardial effusion [7].

Treatment of Lyme-related pericardial effusion primarily involves antibiotic therapy targeting *B. burgdorferi*, with doxycycline or ceftriaxone as the commonly used agents [8]. In most cases, antibiotics resolved the infection and the associated pericardial effusion. Corticosteroids are typically not recommended, as they may impair the body's immune response to the infection. If tamponade is present, urgent pericardiocentesis may be required to relieve the pressure on the heart and restore normal hemodynamics [9]. It is also essential to closely monitor patients for any recurrence of effusion or worsening of cardiac symptoms.

While the majority of Lyme disease patients with pericardial effusion and Lyme carditis respond well to antibiotic therapy, early diagnosis and treatment are critical in preventing complications. Given the rarity of pericardial effusion in Lyme disease, awareness of this potential manifestation is key, especially in regions where the disease is endemic. Clinicians should maintain a high index of suspicion for Lyme disease in patients presenting with unexplained pericardial effusion, particularly in the context of potential tick exposure.

## Conclusions

Pericardial effusion is an uncommon but significant complication of Lyme disease that requires timely diagnosis and treatment to prevent severe outcomes. By recognizing the potential for cardiovascular involvement in Lyme disease, clinicians can improve patient outcomes through early detection and appropriate management.
